# Seed market dynamics and diffusion of new wheat varieties in Bihar, India: a supply-side perspective

**DOI:** 10.1186/s40100-024-00330-w

**Published:** 2024-11-11

**Authors:** Hari Krishnan Kala-Satheesh, Drisya Kuriyedath, Jesna Jaleel, E. P. Nihal Rahman, Archana Raghavan Sathyan, Vijayalaxmi D. Khed, A. G. Adeeth Cariappa, Vijesh V. Krishna

**Affiliations:** 1 Department of Agricultural Statistics, Uttar Banga Krishi Viswavidyalaya (UBKV), Pundibari, Kalarayerkuthi, Cooch Behar, West Bengal 736165 India; 2 Sustainable Agri-Food Systems (SAS) Program, International Maize and Wheat Improvement Center (CIMMYT), #303, ICRISAT Campus, Patancheru, Hyderabad, Telangana 502324 India; 3 Department of Agricultural Economics, College of Agriculture, Professor Jayashankar Telangana State Agricultural University (PJTSAU), Rajendra Nagar, Hyderabad, Telangana 500030 India; 4 Department of Agricultural Extension, College of Agriculture, Kerala Agricultural University, Vellayani, Thiruvananthapuram, 695522 India

**Keywords:** Varietal turnover, Varietal age, Seed demand, Seed supply markets, Private sector breeding

## Abstract

An examination of the dynamics of seed markets in Bihar, India, reveals a paradox—despite an influx of wheat varieties bred by public and private sectors and the proliferation of seed market networks in rural villages, older wheat varieties remain prevalent—necessitating a thorough investigation of the seed distribution system. Unlike most empirical studies that examine the adoption of new and improved crop varieties from a farmer's perspective, our study shifts the focus to the seed supply side. We analyse data collected from 200 private seed dealers who cater to the needs of over 163,000 farmers spread across 10 districts in Bihar. We use descriptive statistics alongside dealer-level and varietal-level regression models to examine the relationship between seed sales and varietal age. Findings indicate that the number of varieties available with a dealer (varietal richness) is positively associated with the number of seed buyers (dealer’s reach) and the total quantity of seeds sold. Private varieties are in demand despite their higher prices. Dealer-level models showed that varietal age affects neither the reach nor the sales, allowing older public-sector varieties to coexist with more recent private-sector ones. However, the varietal-level regression models show that dealers rank the new varieties higher as the ones being sold more. To explore the potential of private seed markets to reduce the proliferation of old wheat varieties that are more susceptible to evolving biotic and abiotic stress factors, we recommend strengthening the varietal registration and seed certification processes, implementing better seed traceability systems, and fostering public–private partnerships in variety development and seed dissemination. Investing in market experiments to incentivize seed dealers to engage in quality assurance can help refine strategies and ensure efficient and inclusive dissemination of promising wheat varieties.

**Supplementary Information:**

The online version contains supplementary material available at 10.1186/s40100-024-00330-w.

## Introduction

Wheat is one of the most important cereal crops in the world, surpassing all other grains in terms of human consumption (Poole et al. 2021; Shiferaw et al. 2013). About 14% of the global wheat supply is contributed by India (FAOSTAT 2022), playing a pivotal role in ensuring food and nutritional security for a significant portion of the global population. However, the country now faces a critical challenge in upholding food security for its rapidly expanding population amid climate change and mounting biotic pressures (Dubey et al. 2020; Bishnoi et al. 2021). Over the past decade, India has witnessed the repercussions of climate change, characterized by a reduction in the duration of winter and the early arrival of summer (Yashavanthakumar et al. 2021). As a result, wheat cultivation faces the adversity of high-temperature stress during the grain-filling stage due to delayed sowing and its proximity to the equator, leading to terminal heat stress (Dubey et al. 2020; Asseng et al. 2015). The increasing temperature adversely affects wheat yield, especially if the crop is late-sown, which is commonly practised in eastern Indian states like Bihar (McDonald et al. 2022; Newport et al. 2020).

Bihar, among the states of India, occupies a significant position in wheat production, ranking sixth in terms of output (Jose and Krishna 2021). This renders wheat cultivation crucial for both the economic stability of farmers and the food security of households (Kishore and Singh 2021). However, wheat production systems in Bihar face several pressing challenges stemming from escalating atmospheric temperatures and the proliferation of pests and diseases. These unfavourable circumstances have severely curtailed the potential of wheat cultivation in the region, thereby jeopardizing food security and the sustainability of agricultural practices (Ramadas et al. 2020). Wheat yields in Bihar not only fall below those of other states in India but also remain considerably lower than the attainable yields (Jose and Krishna 2021).

Recent research underscores the critical role of rapid varietal turnover in enhancing agricultural productivity. Specifically, the adoption of newer, improved varieties has been empirically linked to heightened yield potential, narrowed yield gaps, and better factor productivity, thereby enhancing the resilience of agricultural systems to evolving challenges (Mondal et al. 2016; Pray et al. 2017). Singh et al. (2020) further elaborate on this, suggesting that the pace of varietal replacement across crop-producing regions is positively associated with productivity gains. This assertion is grounded in the premise that newer varieties often embody advancements in genetic resistance to pests and diseases, tolerance to abiotic stresses such as drought and heat, and enhanced nutritional profiles, thereby offering a multifaceted approach to addressing the pressing challenges of climate change and food insecurity. The realization of these benefits, however, is heavily dependent on several factors (Groote and Omondi 2023), including the genetic and agronomic characteristics of the new varieties (Atlin et al. 2017), farmers' access to seed markets (Feder et al. 1985), access to information and support for managing new varieties (Davis et al. 2012), and congenial socio-economic and environmental context (Ishtiaque et al. 2024).

The public sector has dominated wheat varietal development in India since the Green Revolution era and has been releasing several varieties suitable for different agroclimatic zones (Gupta et al. 2018). Recently, many private companies have been involved in wheat breeding and seed multiplication. Nevertheless, despite concerted efforts to promote the adoption of new varieties, the uptake among farmers has been limited, and the average age of wheat varieties in use remains less than ideal (Saroj et al. 2022; Krishna et al. 2016; Jose and Krishna 2021). A small number of mega-varieties, such as PBW 343 and Lok 1, which were released long ago by the public sector and are vulnerable to emerging and intensifying abiotic and biotic stresses, dominate a significant portion of the wheat cultivation area in India. These older varieties may offer some desirable traits, such as superior grain quality (Krishna and Veettil 2022) and could be more affordable for the poor in seed markets. However, in the context of climate change, especially heat stress (Ortiz et al. 2008) and the threat of disease outbreaks like wheat blast (Mottaleb et al. 2019), the sluggish varietal turnover and the continued preference for older varieties form barriers to enhancing agricultural system resilience and achieving sustained productivity growth (Badstue et al. 2022). The literature indicates several farm and system-level factors causing a slow varietal turnover rate in Indian wheat (Pavithra et al. 2017; Saroj et al. 2022). The varietal age and turnover rates are notably lower for women farmers and farmers of socially marginalized communities due to deep-rooted social inequalities (Farnworth et al. 2022; Badstue et al. 2022). Despite these shortcomings in seed delivery to farm gates, few systematic attempts were made to assess the constraints in the seed supply system that allow for the continued prevalence of age-old wheat varieties.

From places of multiplication, crop seeds reach farmers’ fields through multiple pathways, each one differs from others with respect to their institutional structure, approaches and strategies to reach out to a larger clientele (Sisay et al. 2017; Jaleta et al. 2023; Yigezu et al. 2021). A recent study using a regionally representative survey showed that about 83% of wheat farmers in Bihar accessed seeds through private seed dealers (Khed et al. 2024), making them the most crucial players in determining varietal turnover. These dealers sell seeds of varieties released by both the public and private R&D agencies. Such an extensive network of well-established seed companies and retailers could provide an ideal environment for faster varietal turnover and the replacement of obsolete varieties with those possessing higher yield potential, adaptability, and greater resilience to stress. Surprisingly, however, wheat farmers in Bihar continue to buy age-old varieties such as PBW 343 and Lok 1 from private seed retailers (Khed et al. 2024). True, the market also provides some new wheat varieties released by private companies (e.g., Shriram, Ankur, Mahyco, etc.). However, these seeds come at a higher price. More importantly, some of the private retailers are found charging a higher price for seeds of dubious quality (Suri and Gartaula 2023).

In competitive market environments, efficiency is expected to prevail, with effective products like higher-yielding, recent varieties dominating the sales (Rutsaert and Donovan 2020a). However, in the context of Bihar's wheat seed market, we observe a counterintuitive scenario where competition does not straightforwardly lead to the expected efficient outcomes, such as rapid adoption of new varieties. Our observations align with Krishna et al (2016) and Suri and Gartaula (2023), who identify marketing inefficiencies as a significant factor behind the slow spread of new varieties from the public sector in Indian wheat. Despite its importance, the complex interplay between seed supply chain characteristics and the diffusion of improved varieties remains underexplored. Moreover, the predominant role of seed retailers, catering for the needs of most wheat farmers with a diversity of varieties, suggests that seed sales data could offer invaluable insights into varietal prevalence and the impact of private sector involvement in varietal development in partnership with public sector R&D programs.

Against this background, the current paper examines the relationship between varietal age and seed sales (quantity of seeds and number of customers reached) using data from 200 seed dealers from Bihar. The manuscript is structured as follows. The next section reviews the literature on the role of seed supply chains in determining varietal turnover and the adoption of new varieties in agriculture. Sect. "Methodology" details the methodological frame, including study area, sampling framework and empirical framework. The findings of the data analysis are presented and discussed in Sects. "Results and discussion" and "Are the private seed supply chains inclusive?". The last section concludes the study and discusses some policy implications.

## Seed supply chains and technological change in smallholder production systems

Seed supply chains are instrumental in the widespread dissemination of varietal technologies, making improved seed varieties readily available and accessible to farmers. In this section, we review the existing literature connecting the functioning of seed supply networks to varietal turnover and the adoption of new varieties. By taking a supply-side perspective specific to seed, we can better understand the constraints in the varietal diffusion process, and strategies can be developed to effectively connect the demand for new varieties from customers (e.g., farmers, end-consumers, and agro-industry), with the capabilities and requirements of actors in the upstream chain (e.g., seed businesses, traders/retailers, and input and service providers) (Donovan et al. 2021). These supply chains play a crucial role in efficiently producing, distributing, and ensuring the availability of high-quality seeds to farmers, alongside determining the rate of varietal turnover and adoption of new varieties (Gupta et al. 2020). There is an emerging understanding that the relationship between varietal selection and seed source selection was more complex than earlier assumed. For instance, Khed et al. (2024) indicated that farmers’ preference for wheat varieties in eastern India significantly impacted their seed source selection (and not seed sources determining varietal selection, as commonly assumed), indicating a hitherto unexplored causal direction. In the current section, we review the literature connecting the characteristics of seed supply chains to varietal turnover in smallholder production systems of the Global South.

The relevance of seed supply chains rests on their ability to enhance agricultural productivity, which has major effects on the quality, equity, and efficiency of the agri-food systems (Swinnen and Maertens 2007). Plant breeders continuously conduct germplasm improvement R&D and create improved seed varieties with desirable traits like resistance to pests and diseases, tolerance to environmental stress, and higher yield potential (Lantican et al. 2016; Heisey et al. 2003). These innovative varieties have the potential to significantly increase crop productivity, minimize losses from stresses, and thereby impart system resilience. However, the impact of these varieties relies on efficient distribution (Westengen et al. 2023), which is why seed supply chains play a critical role in agrarian development. By effectively delivering these improved seed varieties to farmers, supply chains ensure that farmers have access to the latest innovations and technologies, empowering them to optimize their agricultural output and meet the demands of food production.

When the prevailing supply chains do not deliver the seeds of new varieties that fully meet farmers’ expectations for specific non-yield attributes, such as taste, many farmers persist in cultivating age-old varieties. They express dissatisfaction with formal seed networks' inability to provide quality seeds with desired traits (Krishna and Veettil 2022). In India, these old varieties are often unsupported by upstream R&D entities involved in seed production and policy formulation, including public agricultural departments and research institutions under the umbrella of the Indian Council of Agricultural Research (ICAR), due to concerns about compromising system vigour (Kishore and Singh 2021; Krishna et al. 2016). However, these concerns may not be reflected in the downstream seed agencies (e.g., private seed retailers), which respond directly to farmers’ needs and demands. They provide seeds of popularly demanded old varieties, even though the seed quality and genetic purity may be questionable. As a result, a sub-optimal rate of adoption of new varieties by farmers prevails, necessitating further research into how seed distribution dynamics can be optimized to better serve agricultural productivity and resilience. Further research is needed to understand the nature and effects of incomplete markets and informal seed exchanges, aiming to optimize varietal turnover rates. Additionally, contextualizing farmers' decision-making within socio-cultural and institutional environments is essential when studying varietal adoption patterns (Badstue et al. 2022). The role of contextual factors in shaping technological change has long been recognized, yet only a few studies have empirically addressed this issue, indicating a knowledge gap (Glover et al. 2019).

Furthermore, seed supply chains have a crucial role in upholding seed quality and purity (Barriga and Fiala 2020; Gebeyehu et al. 2019). Proper procedures for seed processing, storage, and transportation are essential to preserve the genetic characteristics and integrity of improved seed varieties. Seed certification programs and quality control measures are implemented within the supply chain to ensure that farmers receive genuine seeds and comply with established standards. This commitment to quality assurance guarantees that farmers can obtain the expected benefits from varietal technologies. By safeguarding seed quality and ensuring faster varietal turnover, supply chains play a pivotal role in the long-term sustainability of agricultural systems, mitigating the risk of crop failure and optimizing overall productivity (Barriga and Fiala 2020). Furthermore, a well-functioning seed supply chain ensures the accessibility and affordability of varietal technologies for farmers, regardless of their geographical location or socio-economic background. This is achieved through the establishment of a network of seed distribution points and the enhancement of transportation infrastructure, addressing logistical hurdles faced by farmers in remote regions. Additionally, economies of scale and market competition within the supply chain can reduce seed prices, making improved varieties more financially viable for resource-constrained farmers. This enhanced accessibility fosters equal opportunities for farmers to adopt varietal technologies, particularly benefiting smallholder farmers and promoting inclusive agricultural development (Westengen et al. 2023).

The nature of seed supply networks determines gender-inclusive rural development to a great extent. Women in developing countries face various obstacles that hinder their participation in seed supply chains, limiting their access to resources, opportunities, and economic empowerment. Traditional gender roles and cultural norms restrict women's decision-making power and control over agricultural resources (Mittal and Hariharan 2021). Discrimination in terms of pay and limited access to training and extension services further marginalize them. Difficulties in accessing land, credit, and modern agricultural technologies also impede their involvement in seed production and distribution (Twyman et al. 2016). Limited market access, inadequate seed storage facilities, and lack of transportation infrastructure make it challenging for women to engage in sustainable wheat production. To promote gender inclusivity, targeted interventions and policy measures are needed to address the specific needs and constraints women farmers face in the Global South (Jaleta et al. 2023).

Finally, seed supply chains not only provide seeds but also contribute to knowledge transfer and capacity building among farmers (Matuschke and Qaim 2008). They serve as a vital conduit for disseminating information and best practices related to varietal technologies (Rutsaert and Donovan 2020b). Stakeholders within the supply chain, such as seed suppliers, extension workers, and other involved parties, offer technical guidance, training programs, and workshops to educate farmers on the benefits and proper utilization of new seed varieties. This knowledge transfer empowers farmers to make informed decisions and adopt appropriate farming techniques, maximizing the potential of varietal technologies and their positive impact on agricultural productivity. By enhancing the capacities of farmers, seed supply chains contribute to the long-term sustainability of agricultural R&D programs.

## Methodology

### Study area

The major crops cultivated in Bihar are cereals: rice, wheat, and maize (Government of Bihar 2015). Rice is the traditional monsoon (kharif) season crop, while wheat has become an important crop of the winter (rabi) season after the Green Revolution. Maize was cultivated during the kharif season, but its cultivation has shifted to the rabi season over the last few decades. Pulses such as mung bean, peas, and lentils are grown mostly in the southern parts of Bihar (Tesfaye et al. 2017). Recent estimates show that Bihar contributes to about 5.8% of India’s wheat grain production from 7.4% of the national wheat area (Government of India 2023). Wheat holds immense significance in the agricultural landscape of Bihar, contributing to the state's food security, socio-economic development, and overall agrarian sustainability (Government of Bihar 2015). Situated in the fertile Gangetic Plains, Bihar boasts favourable agroclimatic conditions characterized by fertile soils, sufficient rainfall, and abundant irrigation water availability, which provide an ideal environment for wheat cultivation (Keil et al. 2017). Despite these favourable factors, wheat yields in Bihar have been below the other wheat-producing states of India over the past decade: about half of those in Haryana and Punjab. One of the major reasons is the low input use by farmers. The cost of cultivation of wheat was in the range of INR 30–40 thousand per hectare in Bihar, which is about half of the cost incurred in wheat production in the higher-yielding northwestern states (INR 60–70 thousand per hectare) (Jose and Krishna 2021).^1^ The lack of infrastructure development and economic incentives contributed to the agricultural stagnation in the state (Kishore 2004).

There are only a few studies that assessed varietal technologies in wheat in Bihar. Among them, Jose and Krishna (2021) indicated that the average age of breeder seeds in the indents for wheat varieties was high (indicating a lack of adoption of new varieties) and increasing over the years. Using expert perceptions on varietal adoption rates, Pavithra et al. (2017) showed that about 75% of the wheat area in Bihar in the 2013–14 season was under four varieties (PBW 343, HD 2733, PBW 502 and PBW 373). Saroj et al. (2022) indicated that while the Bihar Government had been promoting and disseminating newer wheat varieties, farmers were unable to access and adopt them, and the study attributed several farm household attributes as adoption constraints: a lack of formal education and experience, small landholding size and economic backwardness, dependence on informal seed networks, and farmer preference for taste and cooking quality instead of higher yields, to name a few. Recent studies have pointed out the emergence of the private sector in developing (breeding) wheat varieties alongside seed sales in Bihar (Suri and Gartaula 2023; Singh et al. 2019; Khed et al. 2024; Badstue et al. 2022).

### Sampling

**Fig. 1:**
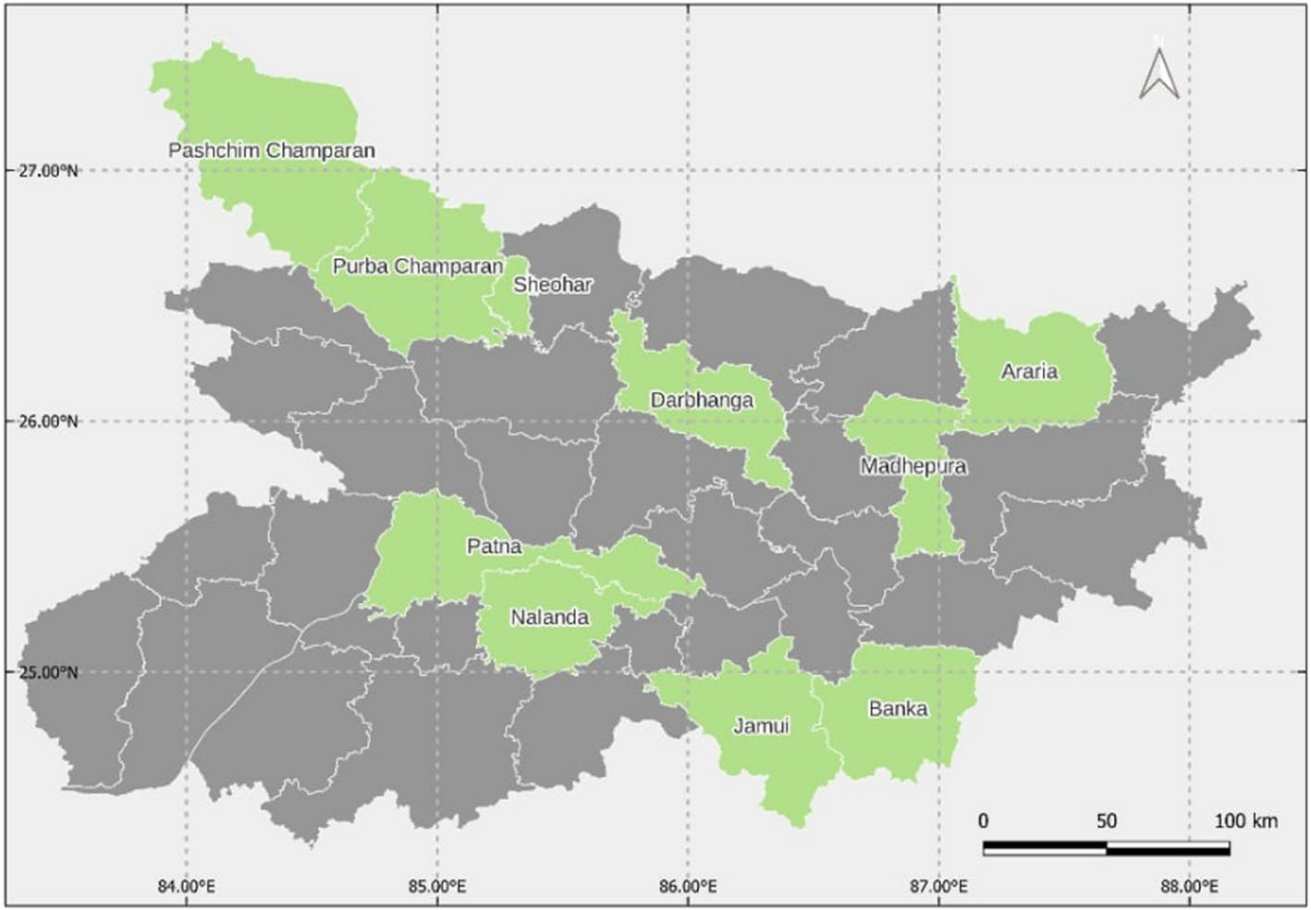
Map showing the study districts of Bihar. *Note*: The districts shaded with light green were included in the study

The study analyses primary data obtained through a face-to-face survey conducted among seed dealers of Bihar, with a structured questionnaire and Computer Assisted Personal Interview (CAPI) approach. The dealer survey was conducted to complement the findings of a farm-household survey carried out among 1000 wheat-growing households in Bihar, and hence, the sampling procedure of the latter must be stated beforehand. For the farm-household survey, a multi-stage random sampling approach was used to select respondents. The first stage was the selection of districts from different agroecological zones of Bihar. Based on soil profile, rainfall, temperature and topography, the state is divided into four agroecological zones (AEZ). Out of Bihar's 38 districts, ten were purposefully selected to guarantee broad representation across the various AEZs, ensuring the inclusion of at least two districts from each AEZ in the study. Specifically, from AEZ 1, Darbhanga, Sheohar, Purba Champaran, and Paschim Champaran were chosen. From AEZ 2, the districts of Araria and Madhepura were selected. In AEZ 3a, the districts of Nalanda and Patna were selected, and from AEZ 3b, Jamui and Banka were included. This selection strategy was employed to ensure a diverse and representative sample of the area under wheat cultivation across Bihar's varied agroecological landscapes. A larger number of districts from AEZ 1 were chosen due to its status as the state's primary wheat-producing region. The study districts are shown in Fig. 1, on the map of Bihar State of India. From the selected districts, four villages each were selected randomly.^2^

For the selection of private seed dealers, a complementary approach was taken. Upon conducting the household surveys and gathering insights into the seed purchasing behaviours of wheat farmers, we identified that a significant majority (approximately 85%) procured their seeds from private dealers. Leveraging this information, we compiled a comprehensive list of seed dealers serving the surveyed villages. From this list, we randomly selected 5 dealers per village for the interviews, ensuring a diverse representation of dealers in terms of geographical distribution and clientele. We carefully monitored the list compilation and avoided any duplication of dealers across the village lists. In rare instances where a villager had reported fewer than five dealers, we compensated by selecting additional dealers from a neighbouring sample village. The dealer survey was conducted among the selected 200 seed retailers in December 2021.

The survey questionnaire covered four key topics: (1) Operational details and variety-specific sales information, (2) Sourcing and timing of seed stock, capturing the procurement process and seasonal sales dynamics, (3) Marketing strategies for new varieties, highlighting dealer efforts in promotion and farmer outreach, and (4) Support services provided to farmers, such as information and credit facilities. The questionnaire used is provided as Online Supplementary Materials S1. We have consulted the local experts (n = 6) working in the field of agricultural extension, plant breeding, and agronomy (two each) to find explanations for some of the unique patterns and surprising findings.

### Empirical framework

The empirical frame was developed keeping the objective of the paper in mind—an exploration of the relationship between varietal age and quantity of seeds sold. When old varieties lose genetic vigour and the production system becomes vulnerable to biotic stresses, farmers would find economic incentives to disadopt them for newer varieties.^3^ If seed dealers effectively interpret and respond to evolving farmer preferences, there is a strong likelihood that they will broaden their inventory to include a diversity of new, high-yielding varieties, aiming to retain their existing clientele and attract new customers (McGuire and Sperling 2013). Alternatively, they could charge a higher price for the new varieties, as compared to the old ones. To estimate the role of varietal age in determining seed supply, two different multivariate regression models were estimated: One at the dealer level, taking the aggregate seed sales as a function of the average age of wheat varieties sold, and the second at the varietal level (fixing dealer characteristics), taking the sales of a given variety as a function of its age. Varietal age is defined as the difference (number of years) between the survey reference year (2021) and the year of official varietal release. Varietal release years were obtained from the websites of wheat breeding institutions and publications (e.g., Gupta et al. 2018). Observations were dropped from the analysis when the varieties carried no information on the year of release. The two types of regression models are detailed below.

In Stage 1, the regression models were estimated at the dealer level (n = 200). There were two models with different dependent variables: the number of buyers per dealer (reach) and the total quantity of seeds sold by a dealer in 2020–21. The dealer characteristics (age, caste, education, experience, etc.) were included alongside the average varietal age, number of varieties sold per dealer and peak time of sale to explain the variation in the dependent variables. Although observed dealer characteristics were controlled in the regression analysis by including them as explanatory variables, there could be unobserved heterogeneities associated with both varietal age and quantity sold (or reach), making the estimates biased. This problem can be avoided in the dealer-level fixed-effects framework.

In Stage 2, the regression models were estimated at the varietal level. Most seed dealers sell more than one wheat variety, and this feature allows us to create variety-level regression models, keeping all dealer characteristics fixed. Here, since we use the variables at the varietal level, averaging of variables (e.g., varietal age) can be omitted. Two types of models were estimated. The first model was with the quantity of sale of seeds per variety as the dependent variable. However, some dealers (31%) were uncomfortable/unwilling to reveal the quantity of the sale of individual varieties, as they feared it would affect their business. The analysis of the quantity of seed sales was carried out with only 69% of the dealer sample. On the other hand, all dealers agreed to rank the varieties sold with respect to the quantity of sale, with the lowest rank given to the variety most sold. Since the data was provided as ranks, an Ordered Probit Model was employed to estimate the role of varietal age in determining seed sales. Here, the dependent variable is ordered and categorical, meaning it has a natural ordering but no fixed numerical interpretation (Greene 2018). Here, the major explanatory variables, other than varietal age, were peak demand date, price of the variety in the previous wheat season and type of the variety (private or public sector origin).

## Results and discussion

Descriptive statistics of key variables from the seed dealer survey are provided in Table 1, which shows significant inter-regional differences. On average, a seed dealer sold wheat seeds to 816 farmers from 19 villages. This average figure masks significant inter-zonal variation—highest in AEZ 2 (1,116 farmers from 23 villages) and lowest in AEZ 3a (579 from 16 villages). While the quantity of seeds sold also showed significant regional differences, it did not follow the same pattern as in the number of buyers. On average, about 227 tons of wheat seeds were sold per dealer in the 2021/22 wheat season. The highest sales were registered in AEZ 2 (728 tons per dealer) and the lowest in AEZ 1 (29 tons). The mismatch between reach and sales across zones is due to the difference in the average quantity of wheat bought per transaction. While the buyers are mostly smallholder wheat farmers, some dealers sell wheat seeds to other distributors in the seed value chains (e.g., smaller shops), and the presence of the wholesale dealers in the sample results in the exceptionally large quantity of seed sold in a single transaction. Most dealers sell varieties of both private and public sector origin, and 35% of dealers were selling at least one variety without a known year of official release.^4^

**Table 1:** Details of the seed sales by sample dealers

	Agro-ecological zones (AEZ) of Bihar				Overall
	AEZ 1	AEZ 2	AEZ 3a	AEZ 3b	
Number of villages catered/covered by the dealer	14.19 (11.20)	23.33 (22.33)	16.06 (11.80)	25.63 (26.47)	18.84^**^ (18.56)
Number of buyers of wheat seeds per dealer per season	722.98 (672.69)	1116.16 (1718.37)	579.44 (1035.62)	888.29 (1827.85)	815.57^***^ (1302.76)
Quantity of wheat seeds sold per dealer per season [tons]	28.57 (40.91)	728.17 (1955.86)	273.45 (1077.30)	49.18 (109.59)	227.29^**^ (1123.89)
Mean value of varietal age [number of years between the year of varietal release and the survey season, i.e., 2021]	17.84 (5.78)	15.39 (7.58)	21.55 (6.10)	18.22 (8.66)	18.06^**^ (7.13)
Number of varieties sold per dealer per season	4.24 (3.39)	2.67 (1.06)	4.47 (1.42)	4.20 (1.29)	3.94^***^ (2.44)
Dealers selling varieties with unknown release year [1 = yes; 0 = no]	0.40	0.28	0.25	0.41	0.35
Dealers selling the wheat varieties released by the private sector only [1 = yes; 0 = no]	0.06	0.34	0.11	0.36	0.19
Dealers selling the wheat varieties released by the public sector only [1 = yes; 0 = no]	0.25	0.02	0.25	0.00	0.15
Average peak date of sales [1 = Nov. 1, 2 = Nov. 2 ……, 61 = Dec. 31]	22.65 (6.91)	23.61 (9.61)	19.63 (9.49)	27.70 (10.19)	23.29 (9.14)
Number of operating hours of the seed shop	10.41 (1.30)	10.49 (1.36)	10.22 (1.22)	10.13 (0.89)	10.34 (1.22)
The shop has a weekly holiday [1 = yes; 0 = no]	0.01	0.33	0.36	0.24	0.19^***^
Dealers selling rice seed also [1 = yes; 0 = no]	0.95	0.98	0.94	0.98	0.96
Dealers selling maize seed also [1 = yes; 0 = no]	0.93	1.00	0.89	0.68	0.89^***^
Dealers selling chemical fertilizers and pesticides also [1 = yes; 0 = no]	0.93	0.84	0.92	0.93	0.91
Dealers obtaining seeds from seed companies [1 = yes; 0 = no]	0.05	0.12	0.03	0.02	0.06
Dealers doing farmer visits to promote varieties [1 = yes; 0 = no]	0.08	0.09	0.08	0.22	0.11^*^
Number of days of village visits for promoting seed sales, conditional on visiting	13.17 (18.57)	10.50 (2.65)	4.33 (3.51)	9.00 (5.59)	9.77 (10.20)
Dealers providing information [1 = yes; 0 = no]	0.98	0.95	0.94	0.98	0.97
Dealers providing input credit to most customers [1 = yes; 0 = no]	0.05	0.12	0.25	0.24	0.14^***^
Dealers providing input credit only to a selected subset of customers [1 = yes; 0 = no]	0.69	0.47	0.44	0.59	0.58^**^
Number of observations (seed dealers)	80	43	36	41	200

Figures in parentheses show the standard deviation of sample mean values. ^***^, ^**^ show that significant regional (inter-AEZ) differences exist at p < 0.01 and p < 0.05, respectively. Kruskal–Wallis' equality-of-population rank test was used for continuous variables, and Pearson’s Chi^2^ test was used for binary variables

According to most seed dealers, the third week of November was identified as the period with the highest wheat seed sales. The timing of peak sales for these dealers showed no significant regional differences. While this peak aligns with the median sowing date in Bihar (last week of November; McDonald et al. 2022), it is considered "late sowing", which can lead to significant yield loss by exposing the crop to terminal heat later in the spring season (McDonald et al. 2022).^5^

One of the key variables of interest, the age of wheat varieties sold by dealers, is also described in Table 1. The mean values of the age of varieties sold by a sample dealer ranged between 15 and 22 years. A sample dealer, on average, was selling 4 wheat varieties. The adoption rate of new varieties was lowest in AEZ 3a (mean varietal age of 22 years) and relatively faster in AEZ 2 (15 years). These average figures are comparable to the existing studies (Khed et al. 2024; Saroj et al. 2022).

Many factors contribute to the observed regional differences in varietal age, including the differences in the prevalence of private sector varieties, most of which are more recently released than the locally popular public sector varieties. For instance, about 34% of seed dealers in AEZ 2 were selling only private sector varieties, which could be the reason for the lower varietal age. In AEZ 3b, a large portion of dealers (36%) sold only varieties developed by private sector seed firms. However, most of these private sector varieties were unregistered, had no recorded year of release, and hence excluded from the varietal age calculations. On the other hand, about 25% of sample dealers in AEZ 1 and 3a were selling only public sector varieties, raising the average varietal age figures of the region.

Seed dealers operated their shops for approximately 10 h daily, with only a slight regional variation. A significant share of dealers observed a weekly holiday, a practice that was more common in AEZ 3a (36% of sample dealers) and least common in AEZ 1 (1%), indicating regional differences in operational practices. Most dealers also sold rice and maize seeds, alongside wheat, with nearly universal coverage across all zones. Additionally, a significant majority offered chemical fertilizers and pesticides, reflecting the diverse agricultural needs catered by these dealers. The most important seed source for the sample dealers was wholesale dealers and direct sourcing from seed companies was limited (6% of dealers only), highlighting a potential gap in the direct distribution chain. Promotional activities, such as farmer visits, were moderately practised, with AEZ 3b dealers being the most active (22% of dealers involved). Most sample dealers indicated that the promotional activities were undertaken by (and were responsible for) the seed company representatives. Information provision to customer farmers was almost universal among dealers, underscoring their role in knowledge dissemination.^6^ The offering of input credit varied. Only a minority of dealers (14%) claimed to provide input credit on wheat seeds to most of their customers, while the majority (58%) provided credit to a selected subset of farmers, suggesting a strategic approach to credit provision based on customer reliability or other factors. Evidence exists in the literature for input dealers employing different credit provision strategies to capture the market (Tittonell et al. 2012).

### Major wheat varieties sold by the sample dealers

Table 2 lists the nine most popular wheat varieties sold by the dealers. Although a total of 43 wheat varieties were available in the market, the remaining 34 were sold only by a few dealers, constituting less than 10% of the total seed sales by volume. Among the top nine varieties, three were released by private companies: Shriram Super 303, Ankur Kedar, and Shriram 252. All of them were recently released, with a varietal age < 10 years. There were six public varieties, namely PWB 343, HD 2967, UP 262, Lok 1, PBW 502, and PBW 373. The most recent variety, HD 2967, was aged 10 years, while others were released several decades ago.

**Table 2:** Major varieties of wheat sold by seed dealers

Varietal name	Year of release	Institution that released the variety	% of Sample dealers selling the variety	% of Dealers ranking this variety with the following ranks:		
				First	Second	Third
Shriram Super 303	2014	Private	84.5%	48.0	14.0	10.5
PWB 343	1995	Public	69.0%	11.5	28.5	22.0
HD 2967	2011	Public	44.5%	17.0	16.0	4.0
UP 262	1978	Public	26.0%	4.5	8.5	9.0
Lok 1	1981	Public	21.5%	9.0	6.5	2.5
Ankur Kedar	2013	Private	20.0%	9.0	8.5	3.0
PBW 502	2004	Public	17.5%	2.0	3.0	3.0
PBW 373	1997	Public	15.0%	1.5	2.5	3.5
Shriram 252	2016	Private	12.0%	1.0	2.5	3.0
Others (with a known varietal age)	–^#^	17.4% private	23.0%	5.5	12.5	10.5
Others (without a known varietal age)	–	93.4% private	46.0%	2.0	3.5	4.0

^#^The weighted average age (standard deviation) of the varietal age is 26.82 (9.64) years. The quantity of sales is used as the analytical weight. Ranks were provided by the dealer based on the quantity of seeds sold

The single most prevalent one was Shriram Super 303, a private variety with a varietal age of seven years, at the time of surveys. About 85% of the sample dealers were selling these varieties, and a large share of them (48%) put it in the first position when ranked with respect to the quantity of seed sold. About 3/4th of dealers included Shriram Super 303 among the top three varieties. According to wheat breeders, the variety Shriram Super 303 is known for its broad adaptability, encompassing yield stability, optimal plant height, and resistance to prevalent diseases such as rusts and Helminthosporium leaf blight. Additionally, its popularity in Central and Peninsular Zones suggests heat tolerance, further underlining its yield stability. The variety's popularity is attributed to these traits, alongside its desired maturity period and bold grain quality, making it a preferred choice across diverse environmental conditions. The second most popular variety was PWB 343, a public variety with a varietal age of 26 years, sold by 69% of the dealers. The third important variety was HD 2967. These varieties were not comparable to Shriram Super 303 with respect to seed sales, but they were included in the top 3 by 62% and 37% of dealers, respectively.

The remaining 34 varieties were categorized into two groups: those with known varietal age and unknown varietal age. Approximately 83% of other varieties with known varietal age were from the public sector. These varieties had a mean varietal age of 26 years. On the other hand, an overwhelming majority (93%) of varieties with unknown varietal age had private sector origins. Only a few dealers (< 10%) included these varieties as the most promising varieties with respect to marketability. Most of these varieties were available only locally, unregistered. The seed experts we interviewed described them as “chameleon varieties”, which could be a public sector variety, named differently to increase seed sales and benefit from the provision of higher prices of private sector varieties.

When varieties with known varietal age were aggregated, the private sector varieties were found to have an average age of eight years and the public sector varieties of 27 years. While the first place with respect to popularity was taken by a private sector variety, the next four positions were occupied by older public sector varieties. This coexistence of new private varieties and old public varieties within the same seed market is highly interesting, contradicting the widely held wisdom that an active seed market could facilitate the dissemination of new, improved varieties (Almekinders et al. 2019; Joshi and Braun 2022).

### Association of varietal age with seed sales

**Fig. 2:**
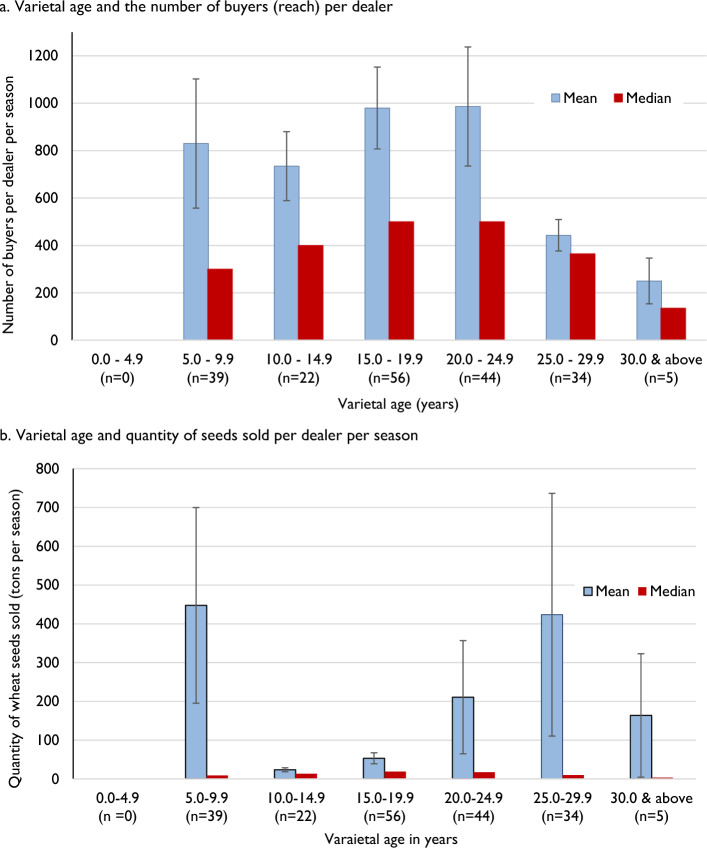
Association between varietal age and seed sales. **a** Varietal age and the number of buyers (reach) per dealer. **b** Varietal age and quantity of seeds sold per dealer per season.  *Note*: Error bars represent the standard error of sample mean values

We capture the sales dimension with two variables: reach or number of buyers and quantity of seeds sold per season. Figure 2a shows that, although 20% of sample dealers were selling varieties aged above 25 years on average, the number of buyers was low. However, when the average age of wheat varieties sold was less than 25 years, there was no correlation of reach with varietal age. Regarding the quantity of seeds sold, both old varieties (25–30 years) and new varieties (5–10 years) had sales exceeding 400 tons per season. In contrast, seeds of varieties aged between 10 and 20 years were sold in quantities of less than 50 tons per season. Although there is greater demand for seeds of varieties aged between 20 and 25 years, the volume sold is only half that of both older (25–30 years) and newer (5–10 years) varieties (Fig. 2b). The outliers were causing a mismatch between the mean and median figures and large standard errors. The median figures values were small in comparison to the mean, denoting a positively skewed distribution. The median sales were associated with varietal age, but the correlation was statistically insignificant. Both Fig. 2a and b indicate no striking evidence for varietal age determining seed sales at the dealer level. Even though the number of buyers was low for very old varieties (> 25 years), they procured seeds in large quantities.

### Association of varietal diversity with seed sales

**Fig. 3:**
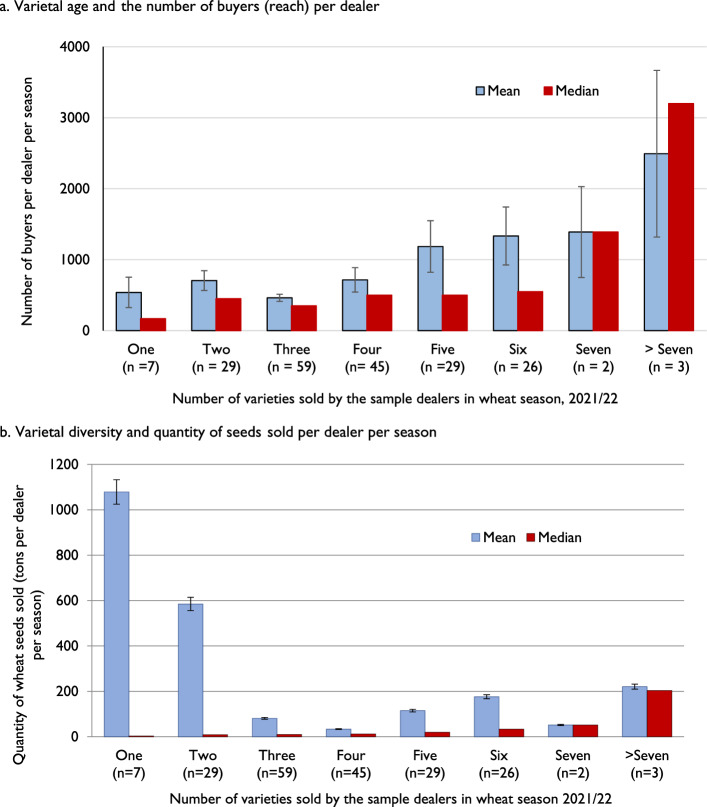
Association between varietal diversity at the shop and seed sales. **a** Varietal age and the number of buyers (reach) per dealer. **b** Varietal diversity and quantity of seeds sold per dealer per season.  *Note*: Error bars represent the standard error of sample mean values

When examining the association between varietal diversity (number of varieties sold by a dealer) and seed sales (quantity of seeds sold and number of customers), we obtained a clear pattern. Figure 3a shows that the dealers who sold a greater number of varieties had a larger number of customers. More than half of the sample dealers sold 3–4 varieties, while approximately 3.5% of dealers offered only one variety. A small fraction, about 2.5% of the dealers in the sample, sold more than six wheat varieties. Interestingly, the dealers selling more than seven varieties had the highest number of buyers (median above 3000 per season). On the other hand, dealers with only one variety had less than 200 buyers (median value). The positive correlation indicates that to reach a larger customer coverage, more varietal diversity might be required in the seed supply chain. Surprisingly, regarding the quantity of seeds sold, a negative correlation exists with respect to the mean values and no correlation for median values (Fig. 3b). The dealers selling a single variety were selling > 1000 tons per season, as per the mean value. Some of the seed dealers in this group could be classified as wholesalers of individual varieties, acting as outliers and resulting in a mean-median disparity.

### Private sector varieties and seed price

**Fig. 4:**
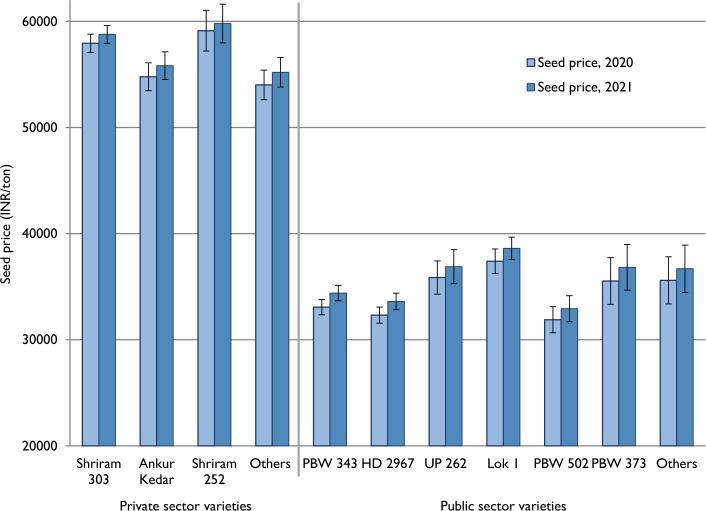
Seed prices of private and public sector varieties. *Note*: Error bars represent the standard error of sample mean values

Most of the wheat varieties sold in Bihar are open-pollinated. Usually, a drastic price difference in the seed market is noticed when open-pollinated varieties coexist with hybrids. However, even in the absence of hybrid wheat, a significant price difference was found to exist between private-sector and public-sector varieties. Figure 4 shows that the selling price of public sector varieties varied between Rs. 30–40 thousand per ton, whereas that of private sector varieties were priced between Rs. 50–60 thousand. Even the seeds of unregistered private sector varieties were fetching more than Rs. 50 thousand per ton. While this unique characteristic of wheat seed markets in Bihar provides a congenial environment for local private firms to develop locally adapted wheat varieties, it also presents a risk of selling public sector varieties after rebranding them as private sector varieties. Among the public sector varieties, the seed price was unrelated to varietal age. For instance, Lok 1 and UP 262, two of the older wheat varieties, seeds of which were sold at a slightly higher price than that of the more recently released variety, HD 2967.

### Effect of varietal age on seed sales: regression analysis

The core of the study is to examine the association between varietal age and seed sales, and here, we use multivariate regression models for this purpose. The seed sales were collected both at the dealer level as the number of buyers and quantity of seeds sold and at the varietal level as the quantity sold. Table 3 shows two regression models at the dealer level. The explanatory variables, alongside mean varietal age, were the number of wheat varieties sold, peak demand date, type of variety (private or public), dealer characteristics (caste, age, etc.), and competition in the local market. The average varietal age was found not significant in determining both the reach and quantity of seeds sold. As seen in Fig. 2a, b, the seed dealers were selling both old-aged and new varieties equally. The total number of varieties sold was positively associated with both reach and the quantity of seeds sold. The number of dealers in the neighbourhood was negatively associated with reach but not with the quantity of seeds sold. There was no statistical significance of most dealer characteristics in the regression model.

**Table 3:** OLS regression estimates on the association between varietal age and seed sales

	Reach [Natural logarithm of the number of buyers per season]		Sales [Natural logarithm of the quantity of wheat seeds sold in tons per season]	
	Coef	(Std. Err.)	Coef	(Std. Err.)
Average varietal age [Years]	− 0.015	(0.015)	− 0.020	(0.022)
Number of wheat varieties sold	0.087^**^	(0.037)	0.140^**^	(0.056)
Average peak date of sales	0.000	(0.011)	0.003	(0.016)
The dealer is selling only private sector varieties [Dummy]	− 0.066	(0.272)	0.291	(0.409)
The dealer is selling only public sector varieties [Dummy]	0.435^*^	(0.265)	0.531	(0.398)
Age of the dealer [Years]	0.011	(0.007)	0.020^*^	(0.011)
Education of the dealer [Years in school]	− 0.005	(0.014)	− 0.005	(0.021)
The dealer belongs to a relatively marginalized caste [Dummy]^#^	− 0.121	(0.217)	0.046	(0.326)
Experience of the dealer in the seed business [Years]	− 0.008	(0.008)	− 0.012	(0.012)
Other inputs (e.g., fertilizers) available in the shop [Dummy]	0.413	(0.291)	0.629	(0.438)
Number of competing seed dealers in the locality	− 0.072^**^	(0.032)	− 0.053	(0.048)
The dealer provides input credits to the buyers	− 0.235	(0.277)	− 0.134	(0.416)
Regional (AEZ) dummies	Included^***^		Included^***^	
*Model statistics*				
Adj. R2	0.14		0.05	
F-Statistic	3.18^***^		1.71^**^	

Here, only one observation per dealer is used

^***^: *p* < 0.01; ^**^: *p* < 0.05; ^*^: *p* < 0.10. Joint significance was considered for the AEZ dummies. Number of observations (dealers): 200. ^#^ There were not many seed dealers belonging to the socially most marginalized caste groups (SC and ST). Here the dummy stands for “Other Backward Castes”, as classified by the Indian Government

In the dealer-level regression models, varietal age values are weighted averages (analytical weight: the quantity of seeds of given variety sold by the dealer in a season), and some of the variation is lost in the averaging process. Also, there could be some unobservable dealer characteristics that affect both varietal age and sales, making the explanatory variable endogenous and estimates biased. To avoid that, we used the provision that most dealers were selling more than one variety. The varietal level regression models, fixing dealer characteristics, were estimated to clearly capture how varietal characteristics affected seed sales. The regression estimates with seed sales as the dependent variable are shown in Table 4. When the dealer-specific variables were not controlled, the varietal age was found to negatively affect sales. One year increase in varietal age was associated with a reduction in seed sales by 2.3%. However, when dealer effects were controlled, the effect of varietal age vanished. The private-sector variety seeds were sold in lower quantities than public-sector varieties, possibly because a relatively smaller number of private-sector varieties are generated by breeders and present in the seed markets. However, the interaction term between private sector variety (dummy) and seed price was positive. For private sector varieties, the increase in seed price was associated with higher sales. The buyers may associate better seed quality with higher prices, resulting in this positive association.

**Table 4:** OLS regression estimates of the association between varietal characteristics and seed sales

Dependent variable: natural logarithm of seed sales (tons/wheat variety/dealer) per wheat season	Without dealer-specific variables included in the model		With dealer-specific variables included in the model	
	Coef	(Std. Err.)	Coef	(Std. Err.)
Varietal age [years]	− 0.023^***^	(0.008)	0.001	(0.006)
Private sector variety [dummy]	− 2.455^***^	(0.758)	− 1.292^*^	(0.694)
Seed price of wheat in the 2020–21 season [INR/ton]	− 3.E-05^***^	(1.E-05)	− 9.E-07	(9.E-06)
Interaction term: Private sector variety x Seed price of wheat in the 2020–21 season	5.E-05^***^	(2.E-05)	3.E-05^*^	(1.E-05)
Peak date of sale	0.007	(0.008)	− 0.021^**^	(0.011)
*Model statistics*				
Adj. R2	0.04		0.59	
F-Statistic	4.95^***^		3.08^***^	

^***^: *p* < 0.01; ^**^: *p* < 0.05; ^*^: *p* < 0.10. Number of observations: 449

Due to the reluctance of some dealers to reveal the variety-specific sales data, we used the ranking of varieties they sell with respect to the quantity sold. The ordered probit model estimates are shown in Table 5. Here, the varietal age was significant and was found to be associated with higher ranks (lower sales). Surprisingly, seed price was associated with lower ranking. Again, higher prices might be linked to better quality by buyers. However, we cannot disentangle the effect of varietal age and price using regression analysis. This is because we have two sets of varieties—highly-priced, recently released private sector varieties and low-priced, old public sector varieties. The absence of high-priced old varieties and low-priced new varieties inhibits us from making meaningful comparisons. More empirical studies using hypothetical varietal traits (e.g., choice experiment) are required to distinguish the role of varietal age on seed market demand.

**Table 5:** Ordered probit regression estimates on the association between varietal characteristics and varietal rank (based on farmer demand)

Dependent Variable: Rank of seed sales (1 = most sold variety), provided by dealers based on farmer demand for seeds	Without controlling dealer effects in the model		After controlling dealer effects in the model	
	Coef	(Std. Err.)	Coef	(Std. Err.)
Varietal age [years]	0.014^***^	(0.006)	0.020^***^	(0.007)
Private sector variety [dummy]	1.137^**^	(0.561)	1.262	(0.914)
Seed price of wheat in the 2020–21 season [INR/ton]	− 1.E-05	(8.E-06)	− 2.E-05^*^	(1.E-05)
Interaction term: Private sector variety x Seed price of wheat in the 2020–21 season	− 2.E-05^**^	(1.E-05)	− 2.E-05	(2.E-05)
Peak date of sale	0.007	(0.006)	0.047^***^	(0.015)
*Model statistics*				
Pseudo R2	0.04		0.15	
LR-Statistic	53.64^***^		175.62***	

Only the first three ranks were included in the regression analysis. ^***^: *p* < 0.01; ^**^: *p* < 0.05; ^*^: *p* < 0.10. Number of observations: 519

### Local expert surveys to validate the empirical estimates

Our study uncovers patterns, particularly the coexistence of private and public sector varieties, that are novel and, to some extent, unexpected. Given the scarcity of research on the wheat seed sector in Bihar, we relied on consultations with local experts to validate our findings after presenting our results and discussing potential explanations for the observed dynamics. Employing local expert surveys to confirm and elucidate research outcomes is acknowledged in academic circles as a reliable method for substantiating empirical observations, particularly in situations where direct empirical or experimental data might be scarce or difficult to gather (Morgan 2013).

The local experts highlighted the possibilities of seed mixing, marketing of inferior quality seeds, and the loss of varietal identity in the seed market of Bihar, especially for seeds without clear brand labels. Some experts have observed that certain dealers multiply seeds from private sector varieties and sell wheat grains as seeds, rebranding them—often as public sector varieties—to avoid litigation. Varietal misidentification is more problematic for public sector varieties, as the dealers face a lower or no risk of litigation. Despite farmers' high valuation of genetic purity and seed vigour, they lack mechanisms to verify these qualities due to the absence of regulatory frameworks. According to local experts, the existing state of confusion, exacerbated by the lack of clear labelling and quality assurance, inadvertently benefits private-sector wheat seed companies. These companies leverage the situation to their advantage, navigating the ambiguities of seed quality and varietal identity more adeptly than their public sector counterparts. The experts opine that this situation is further worsened by the current market and regulatory system, which lacks adequate incentives for seed producers to ensure genetic uniformity and quality. Without a structure that rewards the maintenance of seed quality and penalizes the distribution of substandard products, private seed market agents have little motivation to invest in the rigorous quality control measures necessary for sustaining the genetic purity of wheat varieties. As a result, the efforts to uphold seed quality standards weaken. Therefore, while the private sector is important in introducing new varieties, its impact is mixed, highlighting the need for stricter regulation and quality control. These expert opinions align with the insights derived by Suri and Gartaula (2023), concerning the emphasis on the private sector's dominance in the seed system contributing to the loss of varietal identity and weak distribution channels, which result in the delivery of poor-quality seed to Bihar wheat farmers.

## Are the private seed supply chains inclusive?

We demonstrated the crucial role of the private sector in developing and disseminating wheat varieties within Bihar's seed supply chains, highlighting concerns about excluding farmers from marginalized sections of society in these chains. The relationship between inclusion and privatization can be complex and context-dependent. Previous research has indicated that social norms often hinder women and marginalized communities from participating in input markets, particularly in South Asia (Thorat and Neuman 2010; Raghunathan et al. 2019). Therefore, in this section, we address the issue of inclusivity by examining the proportion of young farmers (aged below 30), women, farmers belonging to marginalized castes (Scheduled Castes or SC and Scheduled Tribes or ST), and marginal landholders (cultivating less than 1 hectare of land under wheat production). The data source is the seed dealer survey, which we have thoroughly explained and referenced at multiple points in this paper. During the survey, the demographic profile of their consumer base was elicited. In addition, the average values from a wheat farmer survey conducted in the same region in the same year (n = 1000 farmers) are provided. Such an examination is highly relevant in light of the Sustainable Development Goals, which emphasize equal opportunities and reduction of inequality in order to ensure that no one is left behind (Mosse 2018). The key findings of this examination are summarized in Table 6.

**Table 6:** Prevalence and access of marginalized sections of the farmer communities to private seed dealers in the study area

	AEZ 1	AEZ 2	AEZ 3a	AEZ 3b	Overall
*Farm survey (2021; % in the sample)*					
Young farmers (aged below 30 years)	7.07 (25.67)	9.41 (29.26)	2.54 (15.77)	3.55 (18.56)	5.95 (23.66)
Farmers from the marginalized castes (SC & ST)	18.43 (38.83)	15.84 (36.60)	35.53 (47.98)	24.37 (43.04)	22.48 (41.77)
Women farmers	11.62 (32.08)	5.45 (22.75)	11.68 (32.19)	10.66 (30.94)	10.18 (30.26)
Marginal farmers (having < 0.40 ha under wheat)	67.42 (46.93)	64.36 (48.01)	56.35 (49.72)	46.19 (49.98)	60.38 (48.93)
*Dealer survey (2021; % among the customers)*					
Young farmers (aged below 30 years)	14.58 (16.01)	17.60 (21.22)	28.34 (25.60)	33.95 (26.67)	22.34 (23.25)
Farmers from the marginalized castes (SC & ST)	19.07 (10.71)	15.82 (11.60)	19.03 (13.20)	24.18 (15.27)	19.50 (12.88)
Women farmers	5.07 (3.84)	5.86 (6.18)	5.31 (4.33)	9.14 (7.14)	6.37 (5.77)
Marginal farmers (seeds bought for < 0.40 ha wheat)^#^	94.76 (3.38)	92.09 (9.45)	92.17 (4.83)	79.22 (14.42)	89.66 (11.08)

The farm survey statistics are used for comparison. This survey was conducted among wheat farmers in the study area, where the sample dealers sell seeds. The number of households covered was 396 in AEZ 1, 202 in AEZ 2, and 197 each in AEZ 3a and AEZ 3b. ^#^The dealers answered this question based on the average quantity of seeds bought. The figures in parentheses show the standard deviation of the sample mean values

### Young farmers

The dealers responded that about 22% of their customers are younger than 30 years of age. In the household survey, the average share of wheat farmers aged below 30 was much lower (6%). There could be two reasons for this mismatch. One, the dealers may be underestimating the age of buyers. Two, the seeds are bought by younger household members, especially when the household head is old. A quick verification of the household survey indicated that about 27.4% of sample households had an additional male member (other than the household head), aged below 30 years, and getting involved in agriculture and allied activities. Many of them will be actively involved in seed procurement, and hence important to be included in the extension programs related to new varieties.

### Marginalized castes

As per the most recent census data, about 17.19% of Bihar's population belongs to SC and ST (Government of India 2019). However, their share in farming could be different; for instance, 6% of operational holdings are managed by SC and ST households in Bihar, which showed a negative trend over time (ibid). Both farm and dealer surveys indicated that about one-fifth of wheat farmers and seed buyers were from these marginalized castes. A notable difference was only in AEZ 3a, where only a smaller share of farmers from marginalized castes accessed seeds from private dealers.

### Women farmers

There are only a small number of women-headed farm households in the study area, and we oversampled the respondents (doubled) in the farm household surveys for comparison with conventional male-headed households. Therefore, the elicited percentage of 6% women customers is close to the reality, although it is lower than the number of female household heads in the farm household survey. More studies are required to be conducted on the reasons and effects of women farmers accessing wheat seed markets in Bihar.

### Marginal landholders

The dealers responded that about 90% of customers buy wheat seeds for < 1 acre (0.40 ha) of wheat. In reality, farmers who cultivate wheat in < 0.40 ha of land are about 60% of the farmer sample population. A possible reason for this disparity is that farmers obtained seeds from more than one source to avoid the risk of spurious seeds. Even if a farmer has > 0.40 ha of land, he may buy a smaller quantity per dealer and may sow farm-saved seeds in a portion of the wheat plot.

Considering the available datasets, there is little evidence of exclusion based on age, caste, and landholding found within the private seed dealer networks. However, the system (and more importantly, farming communities) could be more gender-inclusive, as participation of women in the seed purchases and varietal selection could facilitate that the trait preferences of women would be reflected better. More analysis is required to clearly reflect how women’s participation affects varietal turnover and adoption of improved germplasm. Also, the varietal preferences of young farmers, marginalized castes, and marginal landholders are to be examined, as there could be significant demand heterogeneity (Krishna and Veettil 2022).

## Conclusions and policy implications

The agri-food systems of developing countries face new challenges from climate change and evolving market conditions, to which the varietal portfolio that farmers maintain needs to be adaptable and responsive. To enable farmers to meet evolving requirements and make the system more resilient, the national agricultural R&D programs continue investing in improving crop germplasm. However, the institutional innovations required for delivering these varieties to farmers’ fields, while being equally important, are rather under-invested and under-investigated. Against this backdrop, the present study studied the structure of seed markets in Bihar, which is dominated by private companies and seed dealers, and examined the association between varietal age and seed sales. The uniqueness of private market agents dealing with seeds of varieties released by the public sector R&D, as identified by Tripp and Pal (2001), in addition to the relatively recent emergence of privately bred varieties, points to a dynamic and constantly evolving seed market, the characteristics and implications of which are not sufficiently documented. A major finding of the study is that, despite the high seed price, private-sector varieties are demanded equally as public-sector varieties by wheat farmers. Furthermore, we found only a weak connection between varietal age and farmer demand for varieties. We have also observed that varietal richness at the seed outlet helps the retailers build a consumer base and increase seed sales. Relatively new private and old public sector varieties coexist in the seed market, possibly catering to different market segments. This pattern emphasizes the need for institutional innovations that not only focus on breeding and disseminating new varieties but also ensure that a multitude of varieties suited to the farmers' diverse needs are accessible to all sections of the farming community.

As in the case of wheat breeding, there exist both public and private institutions for seed delivery. The traditional public channels, hurdled with bureaucracy, limited staffing, and ineffective marketing strategies, fail to meet the seed demand of a large share of wheat farmers. On the other hand, various marketing campaigns are organized by private dealers and company personnel (as outlined in Table 1). This strategic approach appears to enable private dealers to effectively reach a substantial proportion (85%) of farmers, even in the most rural areas of Bihar. Furthermore, these dealers play a crucial role in wheat cultivation, as they also act as the source of farming-related information. There could be seed subsidies available in the public seed outlets, but the private seed dealers capture the market with credit for seed and complementary input purchases and are more accessible for farmers. However, the entire market setup currently falls short of adequately incentivizing quality assurance. Drawing from the literature on credence goods, emphasizing the importance of liability and verifiability mechanisms (Kerschbamer and Sutter 2017), the present study recommends investing in innovative market experiments.^7^ The candidate experiments may provide insights into the supply side of Bihar’s seed sector, in particular, the quality assurance challenges and how to align economic incentives to avoid varietal misidentification. The resulting strategies could help enhance the efficiency and inclusivity of seed supply networks. Future research should explore this area.

Strengthening public–private partnerships is another key strategy for broadening access to improved wheat varieties in Bihar. The capacity and potential of Dr. Rajendra Prasad Central Agricultural University in Pusa, Bihar Agricultural University in Bhagalpur, Indian Agricultural Research Institute (IARI) Regional Station in Pusa, Borlaug Institute for South Asia (BISA) Station in Samastipur, and various Krishi Vigyan Kendras (KVKs) in wheat varietal development and seed dissemination signifies the role of public sector in the wheat seed value chains in Bihar. These institutions, by contributing to various aspects of agricultural R&D, including agronomic interventions (e.g., conservation agriculture), efficient input management, and seed systems development, lay a strong foundation for collaborative efforts. Policies encouraging integrating public sector research and breeding capabilities with the private sector's distribution networks could significantly enhance varietal turnover, diversity of varieties in the field, and quality wheat seeds available to farmers. Furthermore, joint variety release programs between public institutions and private seed companies could reduce the incidents of chameleon varieties mentioned earlier in the paper. The strengths of both sectors can be more effectively blended through cooperation, and it will be simpler for farmers to adopt clearly distinguishable varieties.

Although our study has shown how seeds are distributed and which varieties are in demand in Bihar's seed market, there is still much to learn about how farmers decide which seeds to buy. This area deserves further study. Specifically, future research could explore how the differential seed access by different farmer demographics—such as young farmers, women, and those from marginalized communities—is shaped by the local seed market dynamics. Employing methodologies that can capture such demand–supply-side interactions will not only complement the findings of the present study but also enrich our understanding of the process of varietal change.

Alongside exploring avenues for further research, it is important to recognize some limitations of the present study. One key limitation is the potential issue of varietal misidentification, where newer varieties may be sold under different names, potentially compromising the validity of our findings. Moreover, challenges related to seed mixing and the marketing of inferior quality seeds, exacerbated by inadequate quality control measures, raise concerns about the effectiveness of the current seed distribution system in maintaining genetic purity. The study also does not fully explore the decision-making processes of seed dealers, particularly regarding their seed selection, procurement, and understanding of farmer preferences. Addressing these limitations in future research will be essential for developing a more comprehensive understanding of Bihar's seed market dynamics.

Currently, the focus of the R&D programs in the agricultural sector leans heavily towards varietal development, with a significant portion of resources dedicated to breeding and developing new varieties. In contrast, inclusive varietal dissemination—ensuring that these innovations reach the farmers who need them the most—receives comparatively limited funding for innovation. This imbalance highlights a critical gap in the agricultural innovation pipeline, where the end goal of adoption by farmers is not given equal priority. Furthermore, innovative extension approaches, which could bridge the gap between varietal development and farmer adoption, are rarely tested or implemented. This oversight suggests a missed opportunity to enhance the effectiveness of agricultural R&D by ensuring that new varieties are not only released but also adopted by different sections of the farming community.

## Supplementary Information

Supplementary material.

## Data Availability

The data that support the findings of this study are available at CIMMYT Dataverse (https://data.cimmyt.org/). Restrictions may apply to the availability of these data due to privacy concerns. However, aggregated, and anonymized summary data can be provided upon requesting and any further data sharing requests can be directed to the corresponding author [email will be provided after the blind review], after obtaining the necessary approvals and in accordance with applicable data protection and privacy regulations.

## Abbreviations

AEZ: Agro-ecological zone
BISA: Borlaug Institute for South Asia
CAPI: Computer-assisted personal interview
CIMMYT: International Maize and Wheat Improvement Center
IARI: Indian Agricultural Research Institute
ICAR: Indian Council of Agricultural Research
INR: Indian rupee
KVK: Krishi Vigyan Kendra
PPVFR: Protection of Plant Varieties and Farmers' Rights
R&D: Research-and-development
SC: Scheduled castes
ST: Scheduled tribes

## Footnotes

1 1 USD = INR 78.60 in 2022. Source: https://data.worldbank.org/indicator/PA.NUS.FCRF?locations=IN

2 The household survey was conducted to collect data on various aspects of wheat farming in Bihar, including socio-demographic characteristics, plot characteristics, wheat production practices, varietal use, access to seeds, and asset ownership. The survey aimed to understand the adoption patterns and on-farm yield effects of private sector wheat varieties among smallholder farmers in Bihar. The household survey interviews were carried out between August and September 2021. This timing allowed the researchers to gather comprehensive data reflecting the wheat production practices for the 2020–21 Rabi cropping season. In this paper, we are not using the household survey data, except to validate the inclusivity of the private seed sector.

3 The adoption of new varieties is determined by several factors, even outside yield enhancement and profit maximization. While examining the varietal preferences of wheat farmers in Central India, Krishna and Veettil (2022) showed that varietal attribute that ensures grain quality (e.g., *Chapati* taste) was valued with prime importance in the farmer selection of wheat varieties.

4 In India, crop varieties can be marketed without formal registration through the provision for truthfully labelled seeds (Misra et al. 2023), allowing a more flexible approach to seed marketing that accommodates both notified and released varieties. This regulatory flexibility highlights the importance of adaptability in regulations to ensure broader access to various seeds for farmers, thereby supporting the agricultural sector's diverse needs (Kuhlmann and Dey 2021). DNA fingerprinting would be required for accurate identification and parentage. While the Protection of Plant Varieties and Farmers' Rights (PPVFR) Act, 2001, does not explicitly discourage the use of unregistered varieties, it is imperative to note that these unregistered varieties may not benefit from the legal protections and advantages afforded to registered ones(https://plantauthority.gov.in/). Such protections include exclusive rights against infringement, essential for promoting innovation and development within the agricultural sector. Moreover, the Seeds Act of 1966, and its subsequent Amendment Rules play a pivotal role in regulating the quality of seeds for Indian agriculture, mandating the certification of seeds for notified kinds or varieties. Although this Act does not prohibit the use of unregistered varieties outright, it underscores the importance of using certified seeds for farming to uphold crop standards.

5 In Bihar, late sowing of wheat is attributed to several key factors, such as the need to manage the preceding crop cycle, which often includes the harvest and field preparation post-rice cultivation (Ishtiaque et al. 2022). The timing of the monsoon, availability of irrigation resources and access to tillage technologies also significantly influence sowing times (Newport et al. 2020).

6 The input dealers often provide extension and advisory services to market their products, building goodwill and relationships for future business. However, this involvement presents challenges such as capacity issues, conflicts of interest, and coverage gaps. Latha et al. (2021) find that formal training is required to equip input dealers to effectively engage in quality information provision. Seed dealers, through their direct interactions with farmers and participation in local agricultural events, gather valuable insights on emerging trends and preferences. Seed companies also conduct promotional activities and training sessions for dealers, equipping them with information on new seed varieties and their benefits. These sessions, combined with informal networks and discussions with fellow dealers, help seed dealers remain updated on market trends and shifts in farmer preferences.

7 For instance, similar to the experiment conducted by Balafoutas et al. (2013) on taxi drivers’ behaviour towards passengers with varying levels of local knowledge, an experiment could be designed for the seed sector of Bihar. It would entail undercover buyers with differing apparent knowledge levels about seed varieties purchasing from dealers, revealing how informational asymmetry affects dealer behaviour regarding seed quality and pricing. Such an experiment would offer valuable insights into ensuring transparency and quality in the seed market.
