# Supplementary material for: Seed market dynamics and diffusion of new wheat varieties in Bihar, India: a supply-side perspective
Source: Agric Food Econ. 2024 Nov 11;12(1):38. doi: 10.1186/s40100-024-00330-w (PMC11554724; doi:10.1186/s40100-024-00330-w)
Supplement: Supplementary file 1 — Supplementary material. [file 40100_2024_330_MOESM1_ESM.pdf]

Survey Questionnaire

"Informing and Incentivizing the Seed Dealers for Disseminating Improved Varieties"

ENUMERATOR, please introduce the study.

Greetings! I have come as a member of a research team to collect information on wheat variety adoption in Bihar State. This study is conducted by the non-profit organization International Maize and Wheat Improvement Center (CIMMYT). We have previously conducted a farm household survey in 40 villages from 10 districts in 2021. Same as in a lottery, these sample villages were randomly selected from the census of all villages in the district to include in this study. From each village, we asked farmers the names of the main seed dealers providing them with wheat seed. We would like to ask you a few questions on wheat seed market in the region. But before that let us briefly introduce our research work and its implications.

Information is one of the scarcest inputs for crop production in Indian agriculture. While the research organizations are developing new wheat varieties that are more nutritious and resilient to drought and diseases, it has been challenging to bring the seeds to farmers quickly and effortlessly. Because the private dealers of fertilizers, pesticides, and seeds in rural areas often form the major information source, they have high potential to facilitate technology diffusion in rural India. How we can inform and incentivize the dealers to provide information on new resilient and biofortified varieties, forms the research question. The current proposal is for an examination of the willingness of private dealers to act as knowledge dissemination agents – by participating in workshops, demonstration trails, web-based training, etc. – and on different ways to incentivize them to ensure delivery of accurate information and quality seeds of recent wheat varieties with desired traits. The information collected through this survey is important for policymakers, researchers, and other stakeholders for making better decisions towards timely, effective, and equitable delivery of quality wheat seeds in the region. While there are no immediate and visible benefits from participating in this study, your responses would contribute to enhancing agricultural policies and practices from which you may also benefit in the future.

If you participate in this study, we will ask you questions about seed value chains, major wheat varieties, time of seed sales, activities undertaken to reach to a greater number of farmers, etc. It will take about 1.5 hours of your time. You or other household members or your partners in the business will not be exposed to any risks by participating in this study, not be identified personally in any study report or publication. All information about your business will be kept strictly confidential. Data may be shared with other CIMMYT researchers, but we are committed to ensuring absolute confidentiality.

Participation in this study is voluntary. If you choose to participate, you have the right to stop at any time or to not answer individual questions in the questionnaire without any consequences. You may ask any questions that you have about the study to be now or during the interview. If you have questions later, they can be directed to Dr. Vijesh Krishna, Lead Economist (CIMMYT, Hyderabad) by phone at +91 8455 xxxxxx (8am to 4pm) or by email at v.krishna@cgiar.org.

0.1. Did the respondent give his/her consent to be interviewed? ______________ (1= yes/ 0 = no).

If the respondent answered "no", please do not activate the rest of the questionnaire and replace this household with another input dealer.

0.2. If answered yes, interview ID (from the list provided):

____

1 General information

1.1. Seed Dealer Identification

1. GPS coordinates of the shop (If the interview has taken place at dealer's house, please go to the shop and get this coordinates)	*automatically*
2. Date of the Interview	*automatically*
3. Interviewer name (select from the drop-down list)	
4. District name (select from the drop-down list)	
5. Dealer ID (provided to the enumerator)	
6. Dealer name	
7. Shop name, if any	
8. Postal address of the shop	
9. Year of starting of the shop	
10. Owner's name (please write down the full name, including father's name)	
11. Owner's gender (1= female; 0 = male)	
12. Was this shop started by the current owner? (1 = yes; 0 = no; 99 = no idea).	
13. If no, was this shop started by the parents or in-laws of the owner? (1 = yes; 0 = no; 99 = no idea)	
14. Does owner also manage everyday operations and decision-making of the shop? (1 = yes; 0 = no)	
15. Manager's name (please write down the full name, including father's name; *leave the cell blank if manager is the owner*). *The manager should be the respondent of the survey.*	
16. Manager's gender (1= female; 0 = male; *leave the cell blank if manager is the owner*)	
17. Manager's age (years)	
18. Manager's education (*up to which class studied in school; e.g., put 4 if completed 4th standard, 15 if completed degree, 17 if completed masters, 20 if completed PhD*).	
19. Manager's caste (name)	
20. Manager's caste group (1 = SC, 2 = ST, 3 = OBC; 4 = GC)	
21. Is manager a member of any group or union of seed dealers (1 = yes; 0 = no)	
22. If manager is a member, name of the group or union	
23. Number of full-time staff working in this shop now (manager included)	
24. Number of part-time staff working in this shop now	
25. What time does the shop open usually? (e.g., 8am)	
26. What time does the shop close usually? (e.g., 8pm)	
27. Which is the holiday for the shop (e.g., Sunday; multiple options possible)?	
28. From how many villages (number), farmers come to your shop for wheat seed in a year?	
29. What is the average quantity of wheat seeds that a customer buys at a time? (quintal)	
30. Do you also deliver wheat seeds to farmers' house or farms? (1 = yes; 0 = no)	
31. If yes, how many farmers make use of this facility in a year? (number)	
32. Seeds of how many wheat varieties have been sold from this shop in the last season (2021)	
33. Do you sell rice seeds from this shop? (1 = yes; 0 = no)	
34. Do you sell maize seeds from this shop? (1 = yes; 0 = no)	
35. Do you sell chemical fertilizers and pesticides in this shop? (1 = yes; 0 = no)	
36. How many other wheat seed dealers are doing business the neighborhood?	

1.2. Number of farmers buying seeds from the sample villages

Sample village name (*Insert from the list*)	Number of farmers from this village buying wheat seeds in a year (on average)*	Number of farmers from this village buying other inputs in a year (on average)*	Are there any farmer cooperatives or groups buying wheat seeds from you from this village?* (1 = yes; 0 = no)	Are there any women SHGs buying wheat seeds from you from this village?* (1 = yes; 0 = no)
Village 1:_________				
Village 2:_________				
Village 3:_________				
Village 4:_________				
All other villages				

*During the last 3 years. Put 9999 if the dealer is not aware.

2. Wheat varieties and seed sales.

2.1. Would you be comfortable sharing the details of wheat seed sales (quantity figures) in the last (2020) wheat season? ________ (1 = yes; 0 = no).

2.1.1. How many wheat varieties you sold in the last rabi season? (The rows can be added.)

2.2. Please name all wheat varieties, seeds of which are sold from this shop.

Wheat varietal name (Full name)	If 2.1. is yes, please provide the quantity (quintals) of seeds sold for the 2020 wheat (rabi) season	If 2.1. is no, please rank the variety based on seed demand*	Major reasons why farmers go for this variety (Code 1)			Trend in the demand for this variety among farmers over the last 5 years (Code 2)	Seed sale price (Rs/quintal) in		When does the sale of this variety peaks (week and month)?**
			1st	2nd	3rd		in 2020 rabi season	in 2021 rabi season	

* 1 = most demanded for the last (2020) wheat season.

** Example 4th Week of November.

Code 1: 1 = Has high yielding potential (yield) under ideal conditions; 2 = Can provide a certain grain yield in both good and bad weather years (adaptation); 3 = Has high straw yield; 4 = Is taller in height; 5 = Is shorter in height; 5 = Is lodging tolerant; 6 = Is disease resistant; 7 = Has bold grain (grain size); 8 = Has shorter duration (maturity or crop cycle time difference or months or days between sowing and harvesting); 9 = Has longer duration; 10 = Has good tillering capacity; 11 = Requires less seed (seed rate) per unit land; 12 = Can be threshed easily (grains can be separated easily); 13 = Can be stored longer (storability) without damage; 14= Has white grains; 15 = Has yellow grains; 16 = Has brown grains; 17 = High nutrition value; 18 = The dough is more elastic; 19 = Has better fiber content in grain; 20 = Has superior chapati taste; 21 = Has higher demand in the grain market; 22 = others (specify); 23 = Don't know / Can't tell.

Code 2: 1 = New in the market in 2021; 2 = Increasing; 3 = Decreasing; 4 = Steady (no change); 5 = Don't know.

2.3. Did you cultivate wheat for seeds in the last 12 months? _________________ (1 = yes; 0 = no)

2.4. If 2.3 is answered yes, the total wheat area cultivated: _________________ acre.

2.5. How many farmers do you know who cultivate wheat for selling seeds? _____________

3. Rice varieties and seed sales.

3.1. Do you sell rice seeds to farmers? ____________ (1 = yes; 0 = No); If no, please go to section 4.

3.2. Would you be comfortable sharing the details of rice seed sales (quantity figures) in the last (2021) kharif season? ________ (1 = yes; 0 = no).

3.1.1. How many Rice varieties you sold in 2021 kharif season? (The rows can be added.)

3.3. Please name all rice varieties, seeds of which are sold from this shop.

Rice varietal name (Full name)	If 3.2. is yes, please provide the quantity (quintals) of seeds sold for the 2021 Kharif season	If 3.2. is no, please rank the variety based on seed demand*	Major reasons why farmers go for this variety (Code 1)			Trend in the demand for this variety among farmers over the last 5 years (Code 2)	Seed sale price (Rs/quintal) in		When does the sale of this variety peak (week and month)?**
			1st	2nd	3rd		in 2021 season	in 2020 Kharif season	

* 1 = most demanded for the last (2020) wheat season.

** Example <u>4th</u> Week of <u>March</u>.

Code 1: 1 = Has high yielding potential (yield) under ideal conditions; 2 = Can provide a certain grain yield in both good and bad weather years (adaptation); 3 = Has high straw yield; 4 = Is taller in height; 5 = Is shorter in height; 5 = Is lodging tolerant; 6 = Is disease resistant; 7 = Has bold grain (grain size); 8 = Has shorter duration (maturity or crop cycle time difference or months or days between sowing and harvesting); 9 = Has longer duration; 10 = Has good tillering capacity; 11 = Requires less seed (seed rate) per unit land; 12 = Can be threshed easily (grains can be separated easily); 13 = Can be stored longer (storability) without damage; 14= High nutrition value; 15 = Has better fiber content in grain; 16 = Has superior taste; 17 = Has higher demand in the grain market; 18 = others (specify); 19 = Don't know / Can't tell.

Code 2: 1 = New in the market in 2021; 2 = Increasing; 3 = Decreasing; 4 = Steady (no change); 5 = Don't know.

3.4. Did you cultivate rice for seeds in the last 12 months? _________________ (1 = yes; 0 = no)
3.5. If 2.3 is answered yes, the total rice area cultivated in the last year: _________________ acre.
2.5. How many farmers do you know who cultivate rice for selling seeds? _____________

4. Sources of wheat seeds for the dealers

4.1. Number of Sources used for obtaining wheat seeds.

<table>
<tr><th>Please provide the source from which you obtain wheat seeds. Seed source (full name)*</th><th>Seed source type (Code 1)</th><th>% of total seed procured from this source (0-100)</th><th>How many varieties you obtained from this source (Number)</th><th>Name of wheat varieties (full names) obtained from this source in 2020 (add rows to accommodate more varieties)</th><th>Average buying price in 2020 (Rs/quintal)</th><th>The subsidy that you obtained on seed price from the source (Rs/quintal; 0 if no subsidy was obtained)</th><th>Time of procurement (week and month)</th></tr>
<tr><td rowspan="2"></td><td rowspan="2"></td><td rowspan="2"></td><td rowspan="2"></td><td></td><td></td><td></td><td></td></tr>
<tr><td></td><td></td><td></td><td></td></tr>
<tr><td rowspan="2"></td><td rowspan="2"></td><td rowspan="2"></td><td rowspan="2"></td><td></td><td></td><td></td><td></td></tr>
<tr><td></td><td></td><td></td><td></td></tr>
<tr><td rowspan="2"></td><td rowspan="2"></td><td rowspan="2"></td><td rowspan="2"></td><td></td><td></td><td></td><td></td></tr>
<tr><td></td><td></td><td></td><td></td></tr>
</table>

Code 1: 1 = Private wholesale dealer; 2 = another shop/dealer; 3 = State government; 4 = KVK/ University; 5 = BISA; 6 = Own cultivation; 7 = Individual farmers; 8 = Farmer cooperatives; 9 = Company; 10 = Breeder 99 = Others (specify).

* If there are many farmers supplying small quantities of seed (less than 1 ton), indicate them together as “Small farmers”. If a larger farmer is supplying seed (supplying 1 ton or more), please provide his name and village name.

5. Time of wheat seed sales

	Rank the month (period) with respect to wheat seed sales (as per 2020/21 data)*	% of annual sales occurring during this month/ period (as per 2020/21 data)	Most important wheat variety sold during this period (name)
Before August 1st (May, June, July) 2020			
August 2020			
September 2020			
October 2020			
November 2020			
December 2020			
After December 31st 2020 (till May 2021)			

* Rank = 1 when the maximum sales occur (during 2020 and 2021).

6. Activities undertaken to publicize new wheat varieties among farmers.

5.1. Do you visit the villages to inform them about new wheat varieties (though group discussions, exhibiting posters, etc.)? ______ (1 = yes; 0 = no).

6.1. If no, what is the reason for not doing so? [*Please write in respondent's words and go to Q6*].

____

6.2. If yes, do any seed company representatives join you during the village visit? _______ (1 = yes; 0 = no).

6.3. Number of village visits by the seed dealer

	Number of days of village visits for wheat variety publicity
Before August (May, June, July)	
August	
September	
October	
November	
December	
After December (till May)	

6.4. Are you familiar with the varietal promotions carried out by the seed company personnel? _________ (0 = No; 1 = yes).

6.5. Do you think the seed retailers should be actively participated in these promotion programs? _________ (0 = No; 1 = yes).

7. Delivering wheat seed to different farmer categories

6.1. Please rate these groups as the customers of wheat seed.

	Approx. number of farmers visited last 12 months	If they visit, how often do they ask your suggestion on different varieties? (Code 1)
Farmers buying seeds for 1 acre or less.		
Farmers buying seeds for 5 acres or more.		
Women farmers		
Scheduled caste (SC) farmers		
Scheduled tribe (ST) farmers		
Young farmers (age <30 years)		
Old farmers (age >60 years)		

Code 1: 1 = frequently; 2 = sometime; 3 = rarely; 4 = never; 99 = Cannot answer.

7.1. Do you have any strategy (in mind or in practice) to increase your reach among the small farmers (cultivating wheat for 1 acre or less)? If yes, please elaborate (at least 2 lines).

____

____

7.2. Do you have any strategy (in mind or in practice) to increase your reach among the women farmers? If yes, please elaborate (at least 2 lines).

____

____

7.3. Do you have any strategy (in mind or in practice) to increase your reach among the SC/ST farmers? If yes, please elaborate (at least 2 lines).

____

____

7.4. Do you have any strategy (in mind or in practice) to increase your reach among the young farmers (<30 years)? If yes, please elaborate (at least 2 lines).

____

____

8. Information and credit provided

7.1. Do you provide the following service to your customer farmers?

Service	**All** wheat farmers who visit your shop? (Code 1)	For some selected customers? (Code 1)
**Information**		
Information on characteristics of wheat varieties		
Information on arrival of new variety seeds		
Information on which chemicals are effective against the pests or diseases in wheat		
Information on better herbicides		
Information on better fertilizers		
**Input credit**		
Input credit of wheat seeds		
Input credit on chemical fertilizers		
Input credit on herbicides		
Input credit on pesticides		

Code 1: 0 = No; 1 = Yes; 99 = Not applicable.

8.1. If input credits are provided on wheat seeds, what is the average duration of the credit (number of weeks between sale and obtaining the money)?

____

8.2. Do you think targeting women self-help groups (SHGs, like *Jeevika*) with information and input credit would be an effective strategy to disseminate new varieties? _________ (1 = yes/ 0 = no)

8.3. Why?

____

____

9. **Willingness to participate in training programs and demonstrations**

Please read out to the respondent:
CIMMYT or the International Maize and Wheat Improvement Center has been active in India for the last several decades and had played an instrumental role in achieving the Green Revolution. The organization has been working with the national universities (like Rajendra Agricultural University, RAU, of Bihar) and develop several improved wheat varieties over years. A CIMMYT organization called BISA (Borlaug Institute for South Asia, Samastipur) is also actively involved in the delivery of quality seeds to farmers. The recent focus of wheat breeding is development of climate-resilient varieties (drought tolerant varieties, for example) and biofortified varieties (high-zinc varieties, for example). Reaching these varieties to farmers – especially the socially and economically marginalized farmers – quickly is a challenge for all research organizations involved. While some of these new varieties might not be yielding higher than the conventionally popular varieties, they perform well under stress conditions (e.g., drought), provide more nutrient per grain to the consumers, or do both.

8.1. If CIMMYT or BISA is conducting workshops to inform seed dealers and farmers about these new wheat varieties before the wheat season (e.g., in August-September), would you be interested to join?

_________________________________ (0 = no; 1 = maybe; 2 = yes).

8.2. If CIMMYT or BISA is conducting field demonstrations to inform seed dealers and farmers about these new wheat varieties during the wheat season (e.g., in January-February), would you be interested to visit the locations and interact with researchers?

_________________________________ (0 = no; 1 = maybe; 2 = yes).

8.3. Do you expect any kind of incentives (e.g., honorarium to attend the workshop) to increase the chances of your participation, if travel, accommodation, and food are already covered by the center?

_________________________________ (0 = no; 1 = maybe; 2 = yes).

8.4 If incentives are required, please indicate the expectation (type and amount) to attend a one-day workshop in Samastipur, if travel, accommodation, and food are already covered by the center?

____

Please consider the following hypothetical scenario. Suppose the seeds of a particular wheat variety are made available for you. This particular variety yield slightly (say, 5%) lower than the popular wheat variety in Bihar on average. But they have high iron and zinc content, which would benefit those who consume the grains, including farmers themselves.

8.5. Would you be willing to procure these seeds and market them if there is a [__________] % subsidy on the seed price from CIMMYT or other seed sources? *[_________] should be randomly allocated per questionnaire as 0% - 5% - 10% - 15% - 20% - 25%*

_________________________________ (0 = no; 1 = yes).

8.6. If you are willing to procure these seeds to market, how much quantity can be procured in the first wheat season? ____________________ (quintals).

10. Willingness to participate in a CIMMYT project to target the marginalized sections of the society.

As indicated earlier, the CIMMYT researchers have been working to increase the farmer adoption of new, promising wheat varieties among the marginalized section of the society, such as SC/ST farmers, women farmers, farmers with small landholding etc. As part of an ongoing research project, we are considering a technology intervention. Here, we provide the selected farmers with seed vouchers, which can be used to obtain seeds of particular variety free of cost or reduced cost to cultivate in half an acre, <u>through the selected seed dealers</u>. If the project is implemented, CIMMYT will be directly paying (reimbursing) the cost of seeds to the seed dealers. There will not be any risk of financial loss for the dealers. However, the seeds of particular wheat varieties (e.g., High Zinc varieties) will only be covered in this project. The selected dealers will be provided with the details of the project next year, and they may accept or decline the offer to join the project. Can we contact you next year to participate in the study?

___________________________________ (0 = no; 1 = yes).

If not interested, could you please state the reasons?

____

____

End of the interview. Please thank the respondent for his time.

11. Questions to the enumerator.

10.1. Was the respondent comfortable with the questions during the interview? ____________ (1 = always; 2 = sometimes; 3 = rarely; 4 = never).

10.2. Was the shop directly facing the road? ______________ (1 = yes; 0 = no).

10.3. Was the shop easy to find and reach for a new buyer? _______________ (1 = yes; 0 = no).
